# COVID-19 presenting as an exanthematic disease: a case report

**DOI:** 10.1590/0037-8682-0533-2020

**Published:** 2020-09-11

**Authors:** Matheus Todt Aragão, Eusébio Lino dos Santos, Tainah Dantas Ataide, José Seabra Alves, Nathalia Vasconcelos Barroso Todt Aragão

**Affiliations:** 1 Universidade Tiradentes, Departamento de Medicina, Aracaju, SE, Brasil.; 2 Universidade Tiradentes, Departamento de Enfermagem, Aracaju, SE, Brasil.

**Keywords:** COVID-19, SARS-CoV-2, Skin rash

## Abstract

Coronavirus disease (COVID-19) is caused by SARS-CoV-2 and has spread rapidly and caused a global pandemic. Knowledge about clinical and laboratory manifestations in the pediatric population is necessary to guide and monitor such patients. A 3-year-old female patient diagnosed with COVID-19 presented with high fever. After defervescence, she experienced a maculopapular rash that worsened by the sixth day of the disease with self-limited evolution without relevant laboratory changes. The identification of rashes in children with COVID-19 is an unusual and important condition that must be recognized in view of the high transmissibility shown.

## INTRODUCTION

The rapid spread of the coronavirus disease (COVID-19), caused by SARS-CoV-2, has led to a pandemic with infected individuals of all ages and residents of almost all countries worldwide. However, since the first reports, most studies have focused on symptomatic adults. The characterization of clinical manifestations and laboratory findings in the pediatric population is essential to guide the care of such patients to predict disease severity and to determine the prognosis^1^.

Despite the scarcity of samples, children have been identified as an important source of virus spread^8^ ^,^ ^10^. Nevertheless, current papers indicate that children have a lower susceptibility and contagiousness to COVID-19 than adults^2^ ^,^ ^11^.

An assessment of 171 children infected with SARS-CoV-2, treated at Wuhan Children’s Hospital between January 28 and February 26 2020 offers a description of the spectrum of the disease in the pediatric population. Contrary to what has been observed in adults, most children exhibit mild conditions and are often asymptomatic^3^ ^,^ ^9^.

Another review of 72,314 COVID-19 cases by the Chinese Center for Disease Control and Prevention showed that less than 1% of cases occurred among children younger than 10 years^4^. Among these patients, 15.8% showed no sign or symptom of the infection. Regarding clinical manifestations, fever was present in 41.5% of cases during all stages of the disease, with cough (48.5%) and pharyngeal erythema (46.2%) being among the most common signs and symptoms.

Hoang et al. carried out a systematic review with 131 studies published between January 24 and May 11 2020, which comprised 7,780 pediatric patients infected with SARS-CoV-2 from 26 different countries. In that review, 19.3% of patients were described as asymptomatic cases, with fever (59.1%) and cough (55.9%) being the main symptoms. Among other less frequent manifestations, rhinorrhea and nasal congestion (20.0%), asthenia and myalgia (18.7%), and rash (0.25%) were observed^1^.

Skin rashes are characterized by acute, rapidly progressive, usually short-lived erythema. They are usual manifestations of several childhood-related diseases, ranging from infectious, hypersensitive, to indeterminate causes^5^. Viral infections are a major cause of rash in children. However, due to the plurality of etiological agents, it is often difficult to safely establish the diagnosis of a viral rash. The investigation must include all available elements: epidemiological, clinical (dermatological and non-dermatological signs) and biological^5^.

## CASE REPORT

**FIGURE 1:**
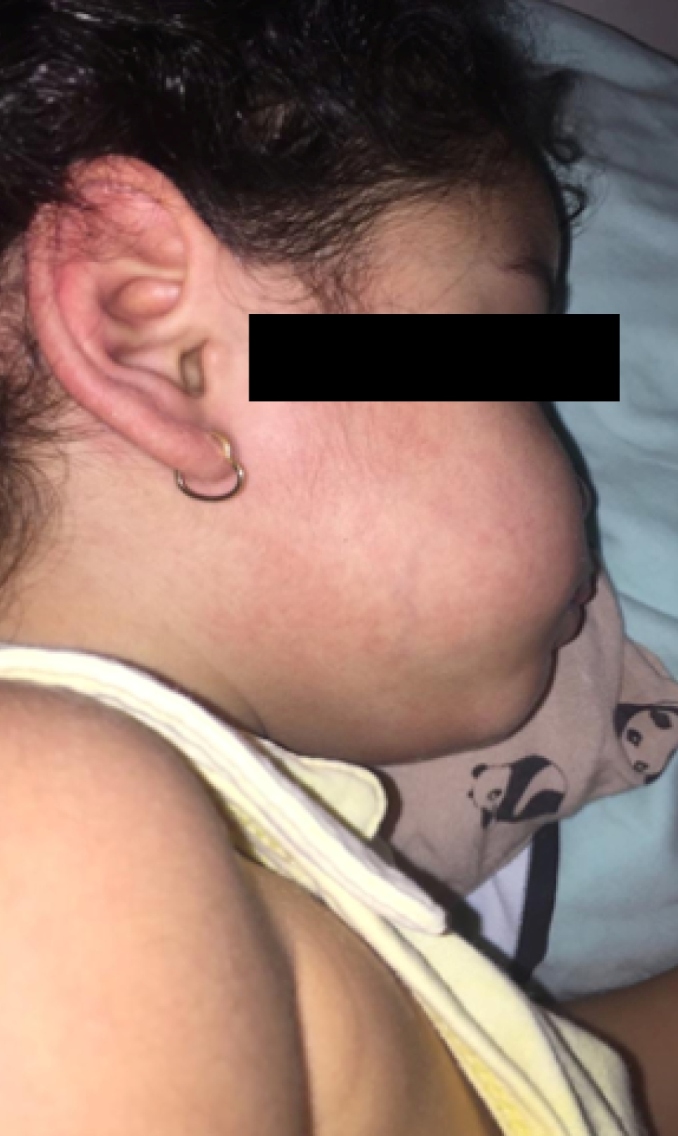
Initial mild maculopapular rash.

A 3-year-old female patient presented with a 3-day history of sudden high fever (temperature 38-39.5°C) associated with severe asthenia. Her mother reported an episode of soft stools without mucus or blood and denied respiratory symptoms. It evolved with defervescence and, shortly thereafter, with the onset of a discreet maculopapular rash and a mild itch (Figure 1).

The patient’s mother reported the absence of relevant morbid history and denied continued use of medications or allergies. The child’s vaccination schedule was up to date. Finally, her mother also denied similar cases among close contacts, recent trips, or contact with sick people.

**FIGURE 2:**
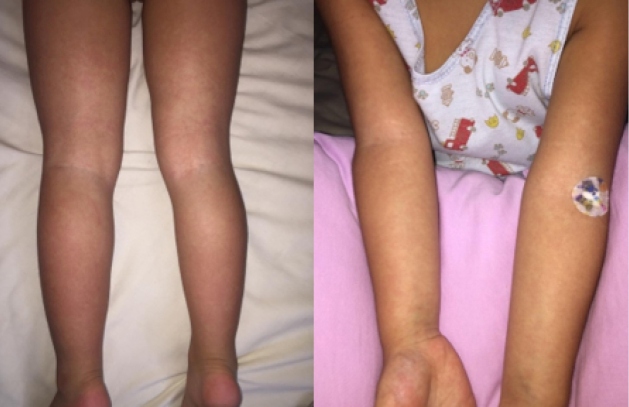
Extensive maculopapular rash on both upper and lower limbs.

Therefore, she sought pediatric medical care which suggested a presumptive diagnosis viral exanthematous disease. She underwent nasal swab collection, to perform RT-PCR for SARS-CoV-2 detection (Charitê Protocol, Bio-Manguinhos) and the medical team prescribed acetaminophen, dipyrone, and desloratadine. The patient’s general condition improved gradually. However, by the sixth day of illness, she experienced a significant worsening of the rash (Figure 2). The RT-PCR assay yielded positive results, without any other relevant laboratory changes (Table 1).

**TABLE 1:** Laboratory test results.

Laboratory tests	Results
Red blood cell count	4.10 × 106/µL
Hemoglobin	11.90 g/dL
Hematocrit	33.60%
White cell count	5,450/mm3
Rods	1%
Neutrophils	37%
Typical lymphocytes	45%
Atypical lymphocytes	8%
Monocytes	4%
Eosinophils	5%
Platet count	231,000/mL
C-reactive protein	<0.5 mg/dL

## DISCUSSION

Children with COVID-19 usually evolve with less severe manifestations, possibly due to the evolution of angiotensin- converting enzyme 2 (ACE2) expression, T cell immunity, and the pro-inflammatory cytokine environment^12^. Another hypothesis is that SARS-CoV-2 competes with other preexisting viruses in the respiratory mucosa of young children^6^.

Patients diagnosed with COVID-19 usually have dermatological manifestations. The pathophysiological mechanisms that potentially explain the cutaneous findings associated with COVID-19 are the SARS-CoV-2 RNA immune hypersensitivity response, cytokine release syndrome, deposition of microthrombi, and vasculitis^6^.

The first report of such cutaneous manifestations was made in an observational study conducted in Italy with a frequency of 20% among hospitalized patients who had had no history of exposure to drugs in the previous two weeks. In that study, 44% of the patients developed cutaneous findings during the initial stage of the disease, while the rest presented during the course of the disease. Among the skin findings, erythematous rashes, urticaria, and vesicles were observed^6^.

Changes in superficial perivascular dermatitis and dyskeratotic keratinocytes were most frequently present on histopathological examination of skin rashes^5^. In some cases, these rashes can be directly related to viral infection, as they occur in other viral diseases such as dengue fever, rubella, and measles^7^.

Skin manifestations are usually self-limiting. It is not possible to state that patients who had cutaneous changes and received biological treatments have a higher risk of complications^6^. The differential diagnosis of exanthematic rash is difficult, as drug eruptions and viral rashes show considerable similarities^7^.

The knowledge that the disease caused by SARS-CoV-2 also produces extrapulmonary repercussions supports the recognition of dermatological manifestations. The pediatric population affected by COVID-19 mostly presents milder symptoms and the appearance of the rash does not prove to be an indicative of severity. Therefore, the identification and differentiation of exanthematic conditions in children resulting from COVID-19, although infrequent, are relevant because this population may represent a source with high transmissibility.
